# Apoptotic signaling pathways in bone metastatic lung cancer: a comprehensive analysis

**DOI:** 10.1007/s12672-024-01151-5

**Published:** 2024-07-26

**Authors:** Yi Zhang, Yi Zheng, Jiakai Zhang, Chaoyang Xu, Junlong Wu

**Affiliations:** 1 Department of Orthopedic Surgery, Ningbo No. 2 Hospital, Ningbo, 315010 Zhejiang China; 2 Health Science Center, Ningbo University, Ningbo, 315211 Zhejiang China; 3 Hangzhou Medical College, Hangzhou, 310053 Zhejiang China

**Keywords:** Apoptosis, Bone metastatic lung cancer, Signaling pathways

## Abstract

This review provides a comprehensive analysis of apoptotic signaling pathways in the context of bone metastatic lung cancer, emphasizing the intricate molecular mechanisms and microenvironmental influences. Beginning with an overview of apoptosis in cancer, the paper explores the specific molecular characteristics of bone metastatic lung cancer, highlighting alterations in apoptotic pathways. Focused discussions delve into key apoptotic signaling pathways, including the intrinsic and extrinsic pathways, and the roles of critical molecular players such as Bcl-2 family proteins and caspases. Microenvironmental factors, such as the tumor microenvironment, extracellular matrix interactions, and immune cell involvement, are examined in depth. The review also addresses experimental approaches and techniques employed in studying apoptotic signaling, paving the way for a discussion on current therapeutic strategies, their limitations, and future prospects. This synthesis contributes a holistic understanding of apoptosis in bone metastatic lung cancer, offering insights for potential therapeutic advancements.

## Introduction

The realm of oncology grapples with the formidable challenge presented by bone metastatic lung cancer, where cancer cells traverse from the primary lung tumor to the bone, inducing a cascade of molecular events [1–3]. This metastatic phenomenon involves intricate alterations in apoptotic signaling pathways, further complicated by the unique microenvironment of the bones that facilitates metastatic colonization [4–6]. The clinical ramifications of bone metastatic lung cancer are profound, significantly contributing to patient morbidity and mortality [7–9]. The clinical impact of lung metastatic bone cancer is substantial, often presenting challenges due to late-stage diagnosis and limited treatment options. Current treatments primarily focus on systemic therapies such as chemotherapy, targeted therapy, and immunotherapy [2, 10]. However, these approaches often yield limited efficacy in advanced stages of the disease. Surgical interventions may be considered for localized lesions, but metastatic spread complicates treatment strategies. Emerging research on apoptotic signaling pathways offers promising avenues for the development of targeted and personalized therapies, providing hope for improved clinical outcomes in the future. A nuanced comprehension of the molecular intricacies governing this metastatic progression is imperative, laying the foundation for the development of precisely targeted therapeutic interventions.

Bone metastatic lung cancer involves a complex interplay of molecular events. The primary lung cancer cells must acquire the ability to invade the surrounding tissue, intravasate into the bloodstream, survive in circulation, extravasate into the bone, and finally colonize the bone microenvironment. This process is facilitated by interactions between cancer cells and the bone microenvironment, including bone marrow stromal cells, osteoclasts, osteoblasts, and immune cells. The bone microenvironment, rich in growth factors and cytokines, creates a fertile ground for metastatic growth.

Apoptosis, a cardinal biological process orchestrating cellular life and death, assumes a pivotal role in the intricate dance of cancer metastasis [11–14]. The dysregulation of apoptosis tilts the delicate equilibrium towards survival, enabling cancer cells to elude programmed cell death and establish proliferative foci at distant anatomical sites [15–17]. In the metastatic cascade, apoptosis stands as a pivotal checkpoint, influencing whether disseminated cancer cells flourish into secondary lesions or succumb to programmed cell death [18–20]. The evasion of apoptosis stands as a hallmark of cancer, and unraveling the modulations of this process during metastasis is of paramount significance [21–23]. Interrogating the apoptotic intricacies in bone metastatic lung cancer not only furnishes insights into the metastatic biology but also unveils avenues for targeted therapeutic interventions aimed at disrupting this precarious balance.

In the metastatic cascade, apoptosis stands as a pivotal checkpoint, influencing whether disseminated cancer cells flourish into secondary lesions or succumb to programmed cell death. The evasion of apoptosis is a hallmark of cancer and unraveling the modulations of this process during metastasis is of paramount significance. Interrogating the apoptotic intricacies in bone metastatic lung cancer not only furnishes insights into the metastatic biology but also unveils avenues for targeted therapeutic interventions aimed at disrupting this precarious balance.

This review seeks to highlight the pivotal role of apoptotic signaling pathways within the complex milieu of lung metastatic bone cancer. By delving into the molecular mechanisms that regulate apoptosis, our aim is to dissect how these pathways critically influence the fate of cancer cells throughout the metastatic process. Gaining an in-depth understanding of apoptotic signaling is crucial, as it helps identify vulnerabilities and adaptive strategies that metastatic cells deploy, thereby informing the creation of precisely targeted therapeutic interventions. The primary objective of this review is to conduct a thorough exploration of apoptotic signaling pathways, with particular focus on their operation and interplay in the context of bone metastatic lung cancer. From the intrinsic mitochondrial pathway to the extrinsic death receptor pathway, we intend to scrutinize the interactions and potential crosstalk occurring within the metastatic lung environment. By examining specific disruptions and modifications in apoptotic pathways linked to bone metastasis in lung cancer, this review aims to contribute valuable insights that are vital for shaping effective and innovative therapeutic approaches.

In essence, this review aspires to bridge the existing knowledge lacuna by seamlessly integrating the research backgrounds of apoptosis, bone metastatic lung cancer, and signaling pathways, presenting a holistic perspective crucial for advancing both academic comprehension and clinical approaches in the ongoing battle against metastatic bone cancer.

## Apoptosis in cancer

### Apoptosis and propagation of bone cancer

Bone metastatic lung cancer intricately weaves a tapestry of molecular events contributing to its aggressive nature and presenting formidable clinical challenges [24–26]. One of the most crucial aspects of this process is how bone cancer cells bypass apoptosis pathways and begin to propagate [27–29]. The dysregulation of apoptotic signaling pathways is a salient feature of this process [29–31].

Cancer cells often upregulate anti-apoptotic proteins such as Bcl-2 and Bcl-XL, while downregulating pro-apoptotic proteins like Bax and Bak [32–34]. This imbalance prevents the activation of the intrinsic (mitochondrial) apoptotic pathway, allowing cancer cells to survive despite significant cellular stress and DNA damage [35, 36]. Inhibitors of apoptosis proteins (IAPs) are frequently overexpressed in cancer cells, inhibiting the activation of caspases, which are essential for executing apoptosis. This inhibition further contributes to the survival and proliferation of bone cancer cells. Activation of survival pathways such as the PI3K/Akt and NF-κB pathways also plays a critical role in bypassing apoptosis [37]. These pathways promote cell survival and proliferation by enhancing the expression of anti-apoptotic proteins and inhibiting pro-apoptotic signals.

Apoptosis, a meticulously regulated programmed cell death process, assumes a pivotal role in cellular homeostasis and holds profound implications within the intricate landscape of bone metastatic lung cancer [24–26]. At its core, apoptosis functions as a precisely orchestrated mechanism that governs the elimination of damaged, mutated, or surplus cells, ensuring the maintenance of tissue integrity and function [27–29].

Fundamentally, apoptosis encompasses a series of highly orchestrated events initiated by intrinsic or extrinsic signals, resulting in morphological and biochemical changes such as cell shrinkage, chromatin condensation, and membrane blebbing [29–31]. Key molecular players, particularly the Bcl-2 family proteins, intricately regulate the delicate balance between pro-survival and pro-apoptotic signals [32–34]. Dysregulation of apoptosis in cancers, including bone metastatic lung cancer, disrupts this delicate equilibrium, enabling cancer cells to evade programmed cell death and persist in hostile microenvironments [35, 36]. In the specific context of bone metastatic lung cancer, an in-depth understanding of apoptosis's fundamental aspects becomes pivotal. The capacity of cancer cells to navigate the metastatic cascade hinges on their ability to evade apoptosis, facilitating the successful colonization of the lungs. Therefore, a comprehensive exploration of the molecular intricacies and regulatory checkpoints governing apoptosis is essential for unraveling the vulnerabilities and adaptive strategies employed by metastatic cells, ultimately informing targeted therapeutic interventions to impede the progression of bone metastatic lung cancer.

### Apoptosis and intravasation

The process of intravasation, where cancer cells invade through the basement membrane into blood vessels, is another critical step in the metastatic cascade that involves evasion of apoptosis. During intravasation, cancer cells undergo Epithelial-Mesenchymal Transition (EMT), a process that confers increased motility and invasiveness [38]. EMT is associated with the downregulation of epithelial markers and upregulation of mesenchymal markers. This transition is also linked to increased resistance to apoptosis, enabling cancer cells to survive in the circulation. Matrix Metalloproteinases (MMPs) degrade the extracellular matrix, facilitating cancer cell invasion into blood vessels [39]. The activity of MMPs is often upregulated in metastatic cancer cells, which also correlates with enhanced resistance to apoptosis. Cancer cells interact with stromal cells, such as fibroblasts and immune cells, within the tumor microenvironment. These interactions can lead to the secretion of survival factors that protect cancer cells from apoptosis during intravasation.

### Apoptosis and niche formation in lung cancer

Once cancer cells reach the lungs, they must establish a metastatic niche, a process that is heavily influenced by apoptotic signaling pathways. To form a metastatic niche, cancer cells must evade the immune system [40, 41]. This is often achieved through the creation of an immunosuppressive microenvironment. Cancer cells can recruit myeloid-derived suppressor cells (MDSCs) and regulatory T cells (Tregs) to the metastatic site, which suppress the immune response and prevent apoptosis.

The formation of new blood vessels is essential for supplying nutrients to growing metastatic lesions [42]. Pro-angiogenic factors such as VEGF are often upregulated in metastatic cancer cells [43]. These factors not only promote angiogenesis but also inhibit apoptosis by activating survival pathways. The interactions between cancer cells and the remodeled extracellular matrix in the lungs are crucial for niche formation. These interactions can activate integrin signaling and other pathways that enhance cell survival and resistance to apoptosis. The lung microenvironment can be hypoxic, which is a stress condition that can induce apoptosis. However, metastatic cancer cells often adapt to hypoxia by upregulating hypoxia-inducible factors (HIFs), which promote cell survival and angiogenesis, thus aiding in niche formation.

## Bone metastatic lung cancer: molecular characteristics

### Molecular features of bone metastatic lung cancer

**Fig. 1:**
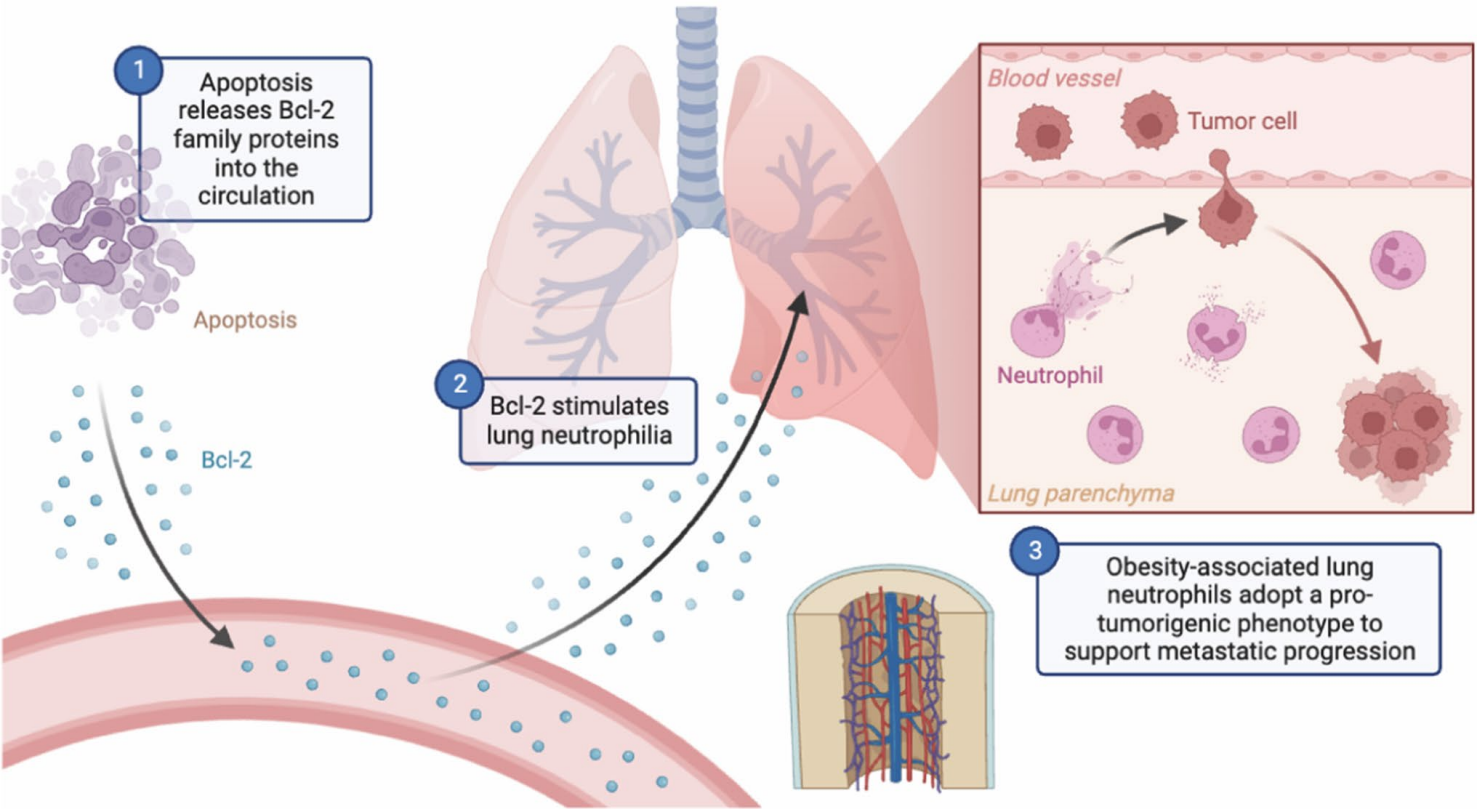
Modulation of lung immune landscape by Bcl-2: a pivotal mechanism supporting bone cancer metastasis. In the intricate landscape of bone metastatic lung cancer, the dysregulation of apoptosis, notably orchestrated by Bcl-2, emerges as a pivotal mechanism reshaping the lung immune landscape to foster metastasis. Bcl-2’s anti-apoptotic influence orchestrates a subtle yet profound alteration in immune dynamics within the lung microenvironment. This orchestrated shift towards apoptosis resistance not only enables cancer cells to persist but also actively subverts immune surveillance. By manipulating apoptotic pathways, particularly through Bcl-2 modulation, bone metastatic lung cancer creates an immunosuppressive niche, providing a sanctuary for metastatic cells to thrive and facilitating their journey from bone to lungs

Bone metastatic lung cancer intricately weaves a tapestry of molecular events, contributing to its aggressive nature and presenting formidable clinical challenges. At the molecular level, the metastatic process unfolds through intricate alterations in signaling pathways, gene expression patterns, and microenvironmental interactions, collectively propelling cancer cells from the primary bone tumor to the lungs. A salient molecular feature defining bone metastatic lung cancer is the dysregulation of apoptotic signaling pathways [44, 45]. Aberrations in both intrinsic (mitochondrial) and extrinsic (death receptor) pathways confer a survival advantage, enabling cancer cells to resist programmed cell death within the metastatic microenvironment [46, 47]. The Bcl-2 family proteins, pivotal regulators of apoptosis, exhibit imbalances favoring anti-apoptotic members, further fortifying resistance to cell death during metastatic progression (Fig. 1).

Moreover, the metastatic journey entails intricate interactions with the extracellular matrix (ECM), a dynamic network of proteins providing structural and signaling cues [48]. Alterations in ECM components, such as matrix metalloproteinases (MMPs), facilitate the invasion of cancer cells into the bloodstream and subsequent migration to the lungs. The immune microenvironment within the lungs undergoes significant molecular changes during metastasis, influencing cancer cells’ ability to evade immune surveillance. Furthermore, molecular heterogeneity is underscored by genetic mutations, epigenetic modifications, and the activation of various signaling pathways, including those implicated in angiogenesis and metastatic colonization [49, 50]. These multifaceted molecular features reflect the dynamic nature of bone metastatic lung cancer, emphasizing the necessity for a comprehensive understanding to inform targeted therapeutic interventions. Unraveling these molecular intricacies holds the key to devising strategies that can disrupt the metastatic cascade, potentially improving outcomes for individuals confronting the challenges posed by bone metastatic lung cancer.

### Mechanisms of apoptosis bypassing and therapeutic resistance in bone metastatic lung cancer

The ability of cancer cells to bypass apoptosis is a critical factor contributing to the development of therapeutic resistance in bone metastatic lung cancer. Apoptosis, or programmed cell death, is a fundamental process designed to eliminate damaged or unneeded cells, thus maintaining cellular homeostasis. In cancer, the dysregulation of apoptotic pathways allows malignant cells to evade this cell death mechanism, leading to unchecked proliferation and survival even in the face of therapeutic interventions.

One of the primary ways in which apoptosis bypassing leads to therapeutic resistance is through the overexpression of anti-apoptotic proteins such as Bcl-2 and Bcl-XL [51, 52]. These proteins inhibit the intrinsic apoptotic pathway by preventing mitochondrial outer membrane permeabilization, a crucial step in the activation of caspases that execute cell death. Consequently, cancer cells with elevated levels of these anti-apoptotic proteins become resistant to therapies that induce cell death via the mitochondrial pathway. Moreover, inhibitors of apoptosis proteins (IAPs) such as XIAP and surviving play a significant role in therapeutic resistance [53, 54]. These proteins directly bind to and inhibit caspases, blocking both intrinsic and extrinsic apoptotic pathways. This inhibition allows cancer cells to survive despite exposure to chemotherapeutic agents or radiation, which typically induce apoptosis through caspase activation. Furthermore, the activation of survival signaling pathways, such as PI3K/Akt and NF-κB, contributes to apoptosis resistance [55, 56]. These pathways promote cell survival, proliferation, and resistance to cell death by upregulating anti-apoptotic genes and downregulating pro-apoptotic genes. Targeted therapies that inhibit these pathways often face resistance as cancer cells adapt by activating alternative survival routes or upregulating compensatory mechanisms.

The tumor microenvironment also supports therapeutic resistance by providing survival signals that protect cancer cells from apoptosis. Interactions with stromal cells, secretion of growth factors, and hypoxic conditions within the bone microenvironment create a niche where cancer cells can evade apoptosis and persist despite treatment. In conclusion, the ability of bone metastatic lung cancer cells to bypass apoptosis is a multifaceted process involving the upregulation of anti-apoptotic proteins, inhibition of caspases, activation of survival pathways, and supportive microenvironmental interactions. This apoptotic evasion is a key contributor to the development of therapeutic resistance, presenting significant challenges for effective treatment.

### Unique challenges in the metastatic microenvironment of bone metastatic lung cancer

The metastatic microenvironment in bone metastatic lung cancer presents a formidable array of challenges that significantly influence the progression and resilience of cancer cells as they traverse from the primary bone tumor to the lungs. Characterized by a dynamic interplay of cellular, molecular, and structural components, this microenvironment shapes the intricacies of the metastatic cascade. A distinctive challenge lies in the interactions with the extracellular matrix (ECM). As cancer cells intravasate and extravasate, encountering a remodeled ECM, dysregulated interactions facilitate dissemination to the lungs [48, 57]. Immune cell dynamics contribute to the complexity, as the immunosuppressive microenvironment allows metastatic cells to establish residence, evading immune surveillance [58]. The intricate interplay between cancer cells and immune components shapes the metastatic microenvironment, influencing both the success of metastatic colonization and potential immune-based therapeutic interventions.

The vascular landscape is pivotal, with angiogenesis supporting the establishment of a vascular network for growing metastatic lesions. Upregulated angiogenic factors, including vascular endothelial growth factor (VEGF), sustain the metabolic demands of proliferating cancer cells [59, 60]. Understanding the unique challenges presented by the metastatic microenvironment in bone metastatic lung cancer is paramount. Insights into molecular and cellular dynamics offer opportunities for targeted therapeutic strategies, potentially disrupting the metastatic cascade and improving clinical outcomes for individuals grappling with the complexities of bone metastatic lung cancer.

## Apoptotic signaling pathways in bone metastatic lung cancer

### A comprehensive exploration of apoptotic signaling pathways in bone metastatic lung cancer

**Fig. 2:**
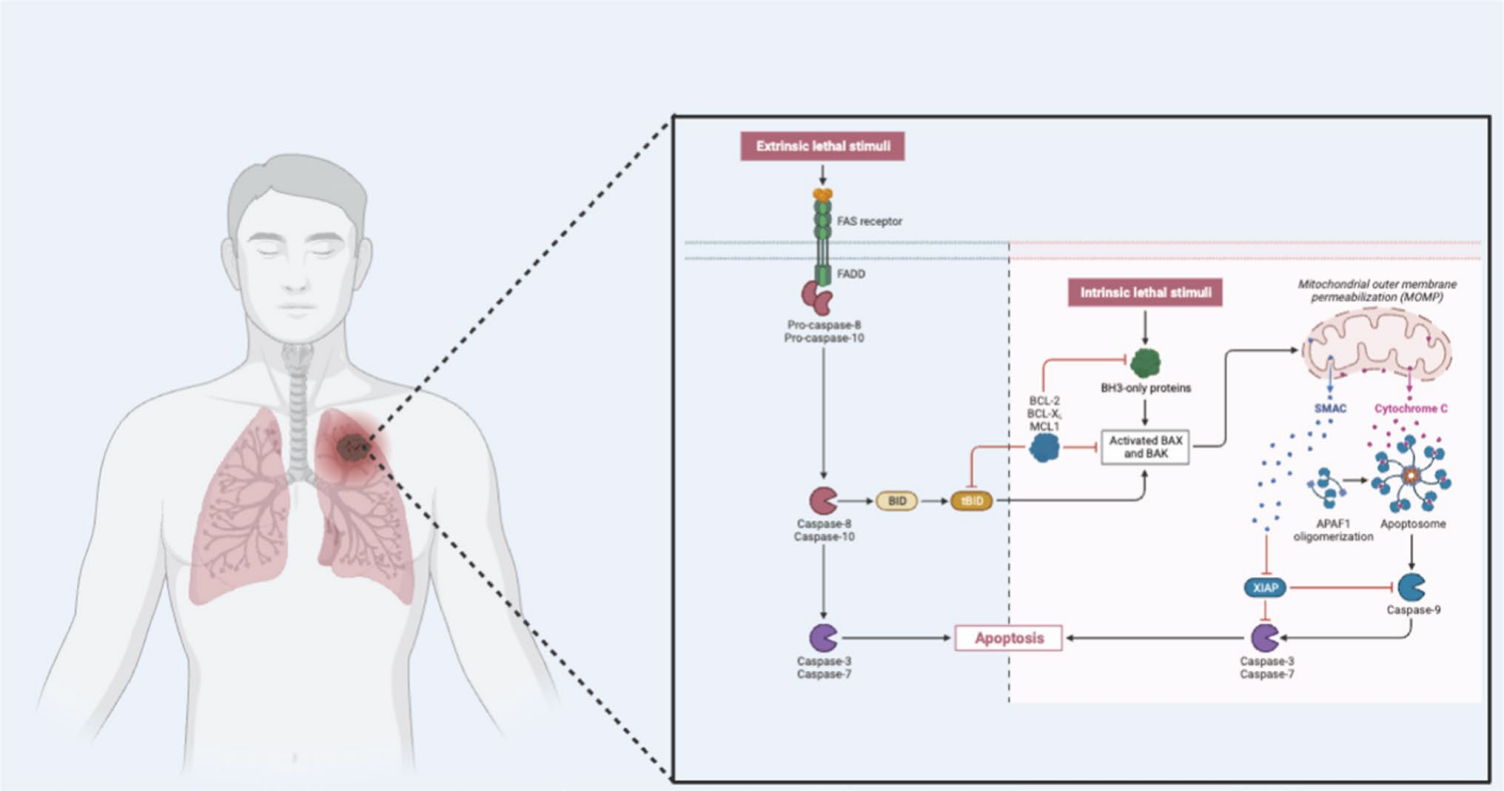
Deciphering Apoptotic Pathways: Unveiling the Intricacies of Evolutionarily Conserved Mechanisms. Apoptosis, a highly sophisticated and evolutionarily conserved process, intricately regulates cellular fate through two fundamental signaling pathways: the intrinsic (mitochondrial) pathway and the extrinsic (death receptor) pathway. The intrinsic pathway responds to intracellular stress signals, triggering mitochondrial events that culminate in programmed cell death. Conversely, the extrinsic pathway is activated by external death signals, initiating a cascade that leads to apoptosis. This delicate interplay between intrinsic and extrinsic pathways exemplifies the evolutionary sophistication of apoptosis, underscoring its pivotal role in maintaining cellular homeostasis and influencing the fate of cells in the context of bone metastatic lung cancer

Apoptosis, an intricately orchestrated and evolutionarily conserved process, is fundamentally steered by two principal signaling pathways: the intrinsic (mitochondrial) pathway and the extrinsic (death receptor) pathway [29, 61]. In the context of bone metastatic lung cancer, a nuanced comprehension of these pathways is indispensable, as their dysregulation profoundly influences the destiny of cancer cells within the metastatic microenvironment (Fig. 2).

#### Intrinsic (mitochondrial) pathway

The intrinsic pathway, intricately connected to mitochondrial regulation, is activated by intracellular stress signals, such as DNA damage or cellular imbalance. In bone metastatic lung cancer, the intrinsic pathway encounters dysregulation, resulting in an imbalance in the Bcl-2 family proteins [62, 63]. Pro-apoptotic proteins, like Bax and Bak, initiate mitochondrial outer membrane permeabilization, releasing cytochrome c into the cytoplasm [64, 65]. Cytochrome c activates caspases, the executioners of apoptosis, culminating in the orchestrated demise of cancer cells. Understanding the nuances of the intrinsic pathway in bone metastatic lung cancer provides insights into vulnerabilities exploitable for therapeutic intervention.

#### Extrinsic (death receptor) pathway

Conversely, the extrinsic pathway is triggered by external signaling through death receptors on the cell surface, such as Fas or TNF receptor [66, 67]. Receptor engagement initiates events activating caspase-8, subsequently leading to downstream effector caspase activation [68–70]. In bone metastatic lung cancer, the extrinsic pathway crucially mediates responses to external stimuli, including interactions with the microenvironment. Dysregulation of death receptor signaling can confer resistance to death receptor-induced apoptosis, contributing to the survival and persistence of metastatic cells.

#### Crosstalk between intrinsic and extrinsic pathways

The crosstalk between intrinsic and extrinsic pathways adds complexity to apoptosis regulation. In bone metastatic lung cancer, intricate molecular interactions exist where signals from one pathway influence the other. For instance, caspase-8, activated in the extrinsic pathway, can cleave Bid, a pro-apoptotic protein translocating to the mitochondria, amplifying the apoptotic signal through the intrinsic pathway [71–74]. Understanding this crosstalk is pivotal, unveiling potential intervention points and highlighting adaptive strategies employed by metastatic cells to evade apoptosis. The intricate interplay between intrinsic and extrinsic apoptotic pathways holds paramount importance in the pathogenesis of bone metastatic lung cancer. While both pathways converge to regulate programmed cell death, their relative contributions to cancer progression remain a subject of intense investigation. The endogenous pathway, primarily regulated by the Bcl-2 family proteins and mitochondrial integrity, plays a central role in sensing cellular stress and initiating apoptosis. Dysregulation of this pathway, characterized by aberrant expression of anti- and pro-apoptotic proteins, can tip the balance towards cell survival, promoting metastatic spread. Conversely, the exogenous pathway, activated by external death signals through death receptors on the cell surface, such as Fas or TNF receptor, orchestrates apoptosis independently of mitochondrial involvement. In bone metastatic lung cancer, the intricate balance between these pathways dictates cellular fate within the metastatic microenvironment. While the endogenous pathway may be more prominently dysregulated due to intrinsic cellular alterations, the exogenous pathway’s activation by factors within the tumor microenvironment, such as immune cells or cytokines, can also significantly influence metastatic progression.

In summation, unraveling the intricacies of apoptotic signaling pathways in bone metastatic lung cancer provides a comprehensive foundation for discerning the molecular underpinnings of cell fate decisions in the metastatic cascade. This knowledge not only enhances our academic understanding but also offers critical insights for the development of targeted therapeutic strategies aimed at restoring the delicate balance of apoptosis in the context of bone metastatic lung cancer.

### Molecular players in apoptosis within the landscape of bone metastatic lung cancer

The regulation of apoptosis in bone metastatic lung cancer hinges on a meticulously coordinated interplay among key molecular players, each contributing to the delicate balance between cell survival and death. Understanding these intricate molecular dynamics is paramount for deciphering the adaptive strategies and vulnerabilities of cancer cells within the metastatic microenvironment.

#### Bcl-2 family proteins

At the forefront of apoptotic regulation stand the Bcl-2 family proteins, a group of critical regulators governing mitochondrial integrity and the intrinsic apoptotic pathway [75]. In bone metastatic lung cancer, alterations in the expression and activity of these proteins play a pivotal role in determining cell fate. The family is divided into anti-apoptotic (e.g., Bcl-2, Bcl-XL) and pro-apoptotic (e.g., Bax, Bak) members [76, 77]. Dysregulation, often marked by overexpression of anti-apoptotic proteins or a shift towards pro-apoptotic counterparts, tips the scales in favor of cell survival, allowing metastatic cells to resist the intrinsic apoptotic pathway [78].

#### Caspases and their activation

Central to the execution of apoptosis are caspases, a family of protease enzymes orchestrating the dismantling of cellular structures. Caspases are categorized into initiators (e.g., caspase-8, caspase-9) and effectors (e.g., caspase-3, caspase-7) [79, 80]. In bone metastatic lung cancer, the activation of caspases serves as the molecular executioner, cleaving essential cellular substrates and driving the cell towards its demise. Dysregulated activation, often influenced by the intricate balance of pro- and anti-apoptotic signals, contributes to the evasion of apoptosis by metastatic cells.

#### Other regulatory proteins

Beyond the Bcl-2 family and caspases, a myriad of other regulatory proteins intricately modulate apoptotic signaling pathways. For instance, inhibitors of apoptosis proteins (IAPs) counteract caspase activity, providing a pro-survival shield for cancer cells. SM-164, an IAPs, effectively eliminated early-stage metastases and hindered the progression of advanced metastatic breast cancer in bone and lung tissues, primarily through TNFα-dependent mechanisms involving IL-4-polarized macrophages. This highlights SM-164’s potential as a promising therapeutic option for triple-negative breast cancer, particularly when administered prior to conventional chemotherapy [81]. Variations in susceptibility to paclitaxel-induced apoptosis were observed among non-small-cell lung cancer cell lines, correlating with the expression of the BH3-only protein, Bim, which proved to be a critical mediator of paclitaxel-induced apoptosis across various cancer cell types, establishing its pivotal role as a molecular link between paclitaxel and apoptosis induction [82]. Additionally, members of the tumor necrosis factor (TNF) superfamily, such as TRAIL (TNF-related apoptosis-inducing ligand), exert influence on death receptor-mediated apoptosis [83, 84]. In this study, engineered mesenchymal stem cells (MSCs) expressing tumor necrosis factor-TRAIL effectively homed to and induced apoptosis in various cancer cell types, leading to significant reduction in tumor growth in xenograft experiments and clearance of lung metastases in a pulmonary metastasis model, highlighting the potential of TRAIL-expressing MSCs as a promising therapeutic strategy for both primary tumors and metastatic disease [85]. In bone metastatic lung cancer, the interplay among these regulatory proteins shapes the cellular response to apoptotic stimuli, influencing the resilience of metastatic cells in the face of intrinsic and extrinsic apoptotic challenges.

Understanding the molecular players in apoptosis within the specific context of bone metastatic lung cancer unveils critical insights into the intricacies of cell fate decisions. This knowledge not only enriches our academic understanding but also lays the foundation for the development of targeted therapeutic strategies aimed at manipulating these molecular players to tip the balance in favor of apoptosis, curbing the progression of metastatic bone cancer.

### Specific alterations in apoptotic pathways unveiled in bone metastatic lung cancer

The pathogenesis of bone metastatic lung cancer is intricately entwined with unique alterations in apoptotic pathways, presenting a distinct molecular landscape that governs the survival and progression of cancer cells within the metastatic microenvironment. In this context, the nuanced dysregulation of apoptotic signaling pathways distinguishes bone metastatic lung cancer from primary bone tumors or metastases to other organs. Alterations in the Bcl-2 family dynamics within bone metastatic lung cancer often manifest as an intricate shift favoring anti-apoptotic proteins, suppressing the intrinsic apoptotic pathway. This imbalance tips the scales towards cell survival, fostering the resilience of metastatic cells in the face of intrinsic challenges. Concurrently, the extrinsic apoptotic pathway, mediated by death receptors, can undergo alterations, influencing the sensitivity of metastatic cells to external apoptotic signals.

Moreover, metastatic adaptation is marked by the modulation of caspase activity. In bone metastatic lung cancer, the dysregulation of caspases may lead to insufficient execution of apoptosis, allowing cancer cells to resist programmed cell death. This altered caspase activity not only influences the dismantling of cellular structures but also contributes to the evasion of apoptotic checkpoints. The intricacies of apoptotic pathway alterations in bone metastatic lung cancer extend beyond well-known players, involving a network of regulatory proteins that collectively sculpt the cellular response to apoptotic stimuli. Understanding these specific alterations at the molecular level provides a foundation for targeted therapeutic strategies. By dissecting the unique apoptotic signatures in bone metastatic lung cancer, researchers can unveil opportunities for precision interventions, ultimately disrupting the adaptive mechanisms employed by metastatic cells and improving clinical outcomes for individuals grappling with this intricate and challenging disease.

## Microenvironmental influences on apoptosis

### Tumor microenvironment and extracellular matrix interactions in bone metastatic lung cancer

The intricate dynamics of the tumor microenvironment (TME) in bone metastatic lung cancer constitute a dynamic and multifaceted milieu profoundly impacting disease progression and therapeutic responses [86]. A convergence of cellular and non-cellular components within the TME orchestrates a complex interplay, fostering the survival and adaptation of metastatic cells. Distinctive alterations characterize the TME in bone metastatic lung cancer, creating a milieu conducive to metastatic colonization and growth [87]. Immune cells within the TME, initially poised for eliminating aberrant cells, assume an immunosuppressive phenotype, affording a protective shield for metastatic cells against immune surveillance [88]. Additionally, the interplay between cancer-associated fibroblasts (CAFs) and metastatic cells emerges as a pivotal factor, influencing the extracellular matrix (ECM) remodeling and creating a supportive niche for metastatic colonization.

The interactions within the ECM in bone metastatic lung cancer stand as a crucial determinant in the metastatic cascade. The process of ECM remodeling plays a crucial role in the progression of both primary and metastatic bone cancer. However, notable distinctions exist between the ECM alterations observed in primary bone tumors and those occurring in metastatic lesions. In primary bone cancer, such as osteosarcoma or chondrosarcoma, the ECM undergoes extensive remodeling characterized by aberrant deposition of collagen, proteoglycans, and glycoproteins, contributing to the aggressive growth and invasion of the tumor. Conversely, in metastatic bone cancer originating from lung primaries, the ECM remodeling process is influenced by both the intrinsic properties of the metastatic cells and the microenvironment of the bone tissue. Metastatic cells, upon homing to the bone, interact with the pre-existing ECM, triggering further remodeling processes. This interaction often leads to the deposition of osteolytic factors such as RANKL (Receptor Activator of Nuclear Factor Kappa-B Ligand), which promotes osteoclast-mediated bone resorption, creating a supportive niche for tumor growth. Additionally, metastatic cells may secrete factors that alter the composition and stiffness of the ECM, facilitating their colonization and survival within the bone microenvironment. Beyond its structural role, the ECM serves as a dynamic reservoir of signaling molecules modulating cellular behavior. Throughout the metastatic journey, cancer cells intricately engage with a remodeled ECM, facilitating invasion, intravasation, and extravasation. Proteolytic enzymes, including matrix metalloproteinases (MMPs), contribute to ECM remodeling, enabling cancer cells to breach the basement membrane and traverse the bloodstream. Upon reaching the lungs, the altered ECM shapes the fate of metastatic cells, providing cues that either promote or impede their colonization and subsequent growth. Understanding this intricate interplay between tumor cells and the ECM within the context of bone metastatic lung cancer offers crucial insights into the molecular mechanisms underpinning metastatic progression, presenting potential targets for therapeutic interventions aimed at disrupting the metastatic cascade.

### Immune cell involvement in bone metastatic lung cancer

Immune cell participation in bone metastatic lung cancer intricately molds the dynamic interplay between metastatic cells and the host’s defense mechanisms. Within the tumor microenvironment (TME), immune cells play a dual role, acting as both sentinels and facilitators of metastatic progression. Despite initial immune surveillance aiming to eliminate circulating metastatic cells, the TME often fosters an immunosuppressive milieu, enabling metastatic cells to elude immune detection and elimination. Frequently recruited myeloid-derived suppressor cells (MDSCs) and regulatory T cells (Tregs) exert immunosuppressive effects, contributing to immune escape [89, 90]. Furthermore, the dynamic interactions between metastatic cells and immune cells within the lung microenvironment influence the fate of disseminated cancer cells, modulating the balance between immune-mediated control and escape. The emergence of immunotherapies has revolutionized cancer treatment paradigms, offering promising avenues for addressing lung metastatic bone cancer. Immunotherapeutic strategies, such as immune checkpoint inhibitors and adoptive cell therapies, harness the power of the immune system to recognize and eradicate cancer cells. Importantly, these immunotherapies have intricate interactions with apoptotic pathways, influencing both tumor cell survival and immune cell function within the tumor microenvironment. For instance, immune checkpoint inhibitors targeting programmed cell death protein 1 (PD-1) or its ligand (PD-L1) can enhance the anti-tumor immune response by blocking inhibitory signals that dampen T cell activity, thereby promoting apoptosis of cancer cells. Additionally, adoptive cell therapies, such as chimeric antigen receptor (CAR) T cell therapy, involve engineering T cells to express receptors targeting tumor-specific antigens, leading to the apoptotic killing of cancer cells upon recognition. Furthermore, emerging evidence suggests that apoptotic pathways play a role in regulating immune cell function and survival.

Angiogenesis, the hallmark of cancer progression involving the formation of new blood vessels, assumes a pivotal role in the growth and sustenance of metastatic lesions in the lungs in the context of bone metastatic cancer. In bone metastatic lung cancer, the angiogenic switch is activated, prompting the secretion of pro-angiogenic factors such as vascular endothelial growth factor (VEGF) and fibroblast growth factor (FGF) [59, 91]. These factors stimulate the sprouting of new blood vessels, providing a nutrient-rich and oxygenated microenvironment for metastatic cells to thrive. Active participation of vascular components, including endothelial cells and pericytes, in the creation of this vascular network is crucial. Furthermore, the intricate crosstalk between metastatic cells and vascular components within the lung microenvironment influences metastatic potential and dictates the angiogenic phenotype. A nuanced understanding of angiogenesis and vascular interactions in bone metastatic lung cancer is essential for identifying potential targets for therapeutic intervention, offering avenues to disrupt the supportive vascular network and impede the relentless growth of metastatic lesions in the lungs.

## Therapeutics and combination therapy protocols targeting apoptosis in bone metastatic lung cancer

### Emerging therapeutics and combination strategies for overcoming bone metastatic lung cancer

Recent advancements in cancer therapeutics have increasingly focused on targeting apoptotic pathways to overcome bone metastatic lung cancer, aiming to counteract the mechanisms that cancer cells use to evade programmed cell death (Table 1). This strategy is crucial in addressing the therapeutic resistance that often complicates the treatment of metastatic cancers.

**Table 1:** Recent therapeutics and combination therapy protocols that target apoptosis to overcome bone metastatic lung cancer

Therapeutic approach	Mechanism of action	Examples	Combination strategies	Advantages
BH3 Mimetics	Mimic action of pro-apoptotic BH3-only proteins, inhibit anti-apoptotic Bcl-2 family proteins	Venetoclax (ABT-199)	Chemotherapy, Tyrosine Kinase Inhibitors (TKIs)	Restores apoptotic process, synergistic effects with other therapies
TRAIL Receptor Agonists	Activate extrinsic apoptotic pathway via death receptors	AMG 655, Dulanermin	Chemotherapy, Radiation, Immune Checkpoint Inhibitors	Selectively induces apoptosis in cancer cells, enhances therapeutic efficacy
Immune Checkpoint Inhibitors	Block inhibitory signals to reactivate immune responses against cancer cells	Pembrolizumab (anti-PD-1), Nivolumab (anti-PD-1)	BH3 Mimetics, TRAIL Agonists	Enhances immune-mediated apoptosis, reactivates immune response
Nanoparticle-Based Drug Delivery Systems	Enhance delivery and bioavailability of apoptosis-inducing agents, minimize systemic toxicity	Various nanoparticle formulations	Combination of therapeutic agents targeting multiple pathways	Multifaceted attack on cancer cells, improved delivery and reduced toxicity

**Fig. 3:**
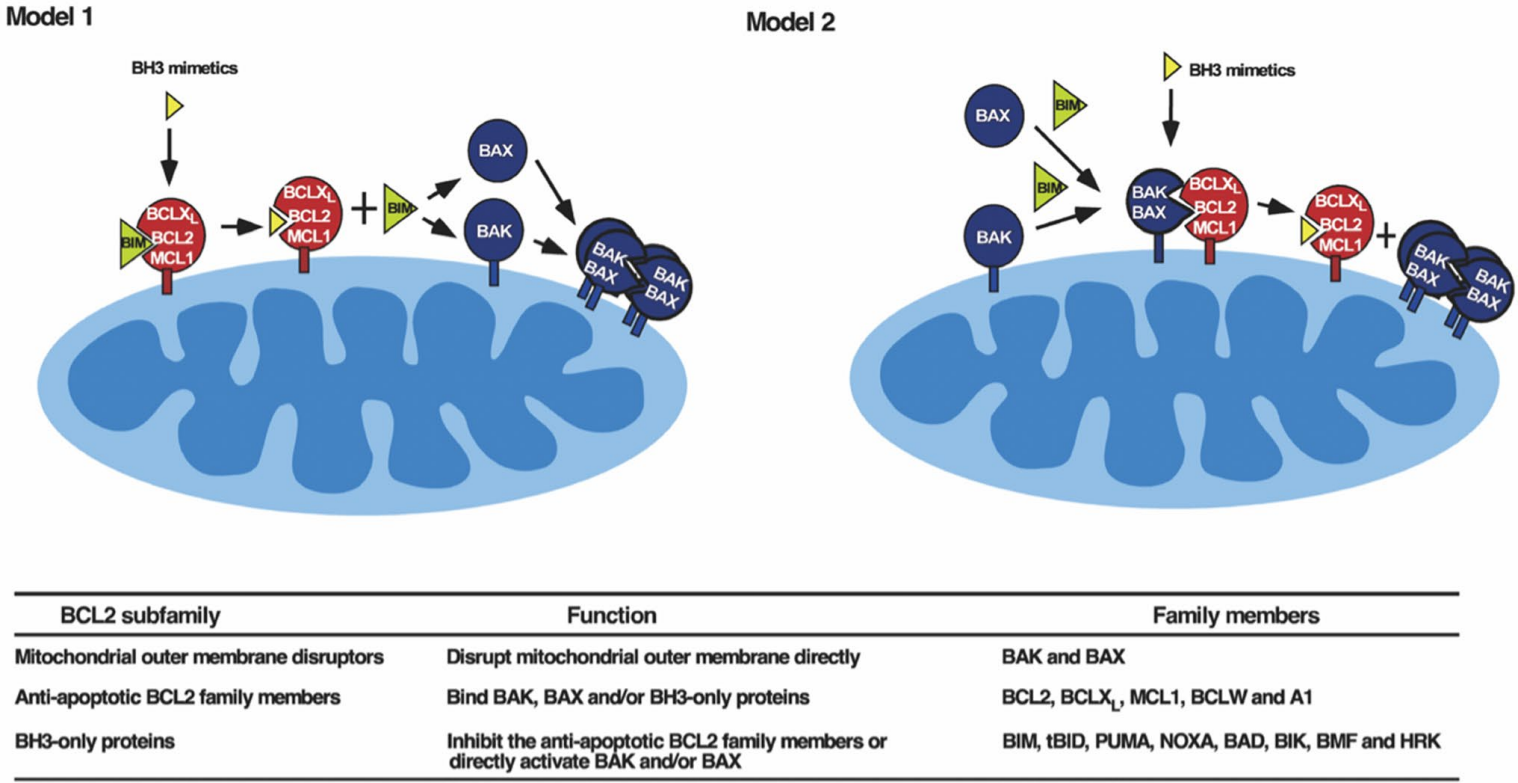
Two models of BH3 mimetic action. Model 1 (left): BH3 mimetics are thought to displace activated BIM from anti-apoptotic BCL2 family members. This displacement allows BIM to subsequently activate BAX and BAK. Model 2 (right): BAK and/or BAX are constitutively activated and are displaced from anti-apoptotic BCL2 family members by BH3 mimetics. This model is more compatible with recent studies showing that BAK and BAX can be activated in the absence of BH3-only proteins under cell-free conditions and in gene-targeted HCT116 cells

One promising approach involves the use of BH3 mimetics, a class of drugs designed to mimic the action of BH3-only proteins, which are pro-apoptotic members of the Bcl-2 family [92, 93] (Fig. 3). These drugs, such as venetoclax (ABT-199), selectively inhibit anti-apoptotic proteins like Bcl-2, Bcl-XL, and Mcl-1, thereby restoring the apoptotic process in cancer cells [94, 95]. Clinical trials have shown that BH3 mimetics can effectively induce apoptosis in various cancer types, including hematologic malignancies and solid tumors, by releasing the inhibition on pro-apoptotic proteins like Bax and Bak, leading to mitochondrial outer membrane permeabilization and subsequent caspase activation. Combining BH3 mimetics with other therapeutic agents has shown enhanced efficacy. For example, the combination of venetoclax with chemotherapy or targeted therapies, such as tyrosine kinase inhibitors (TKIs), has demonstrated synergistic effects in preclinical and clinical studies [96, 97]. This combination leverages the apoptotic sensitization provided by BH3 mimetics with the direct cytotoxic effects of chemotherapy or the inhibition of survival pathways by TKIs [98].

**Fig. 4:**
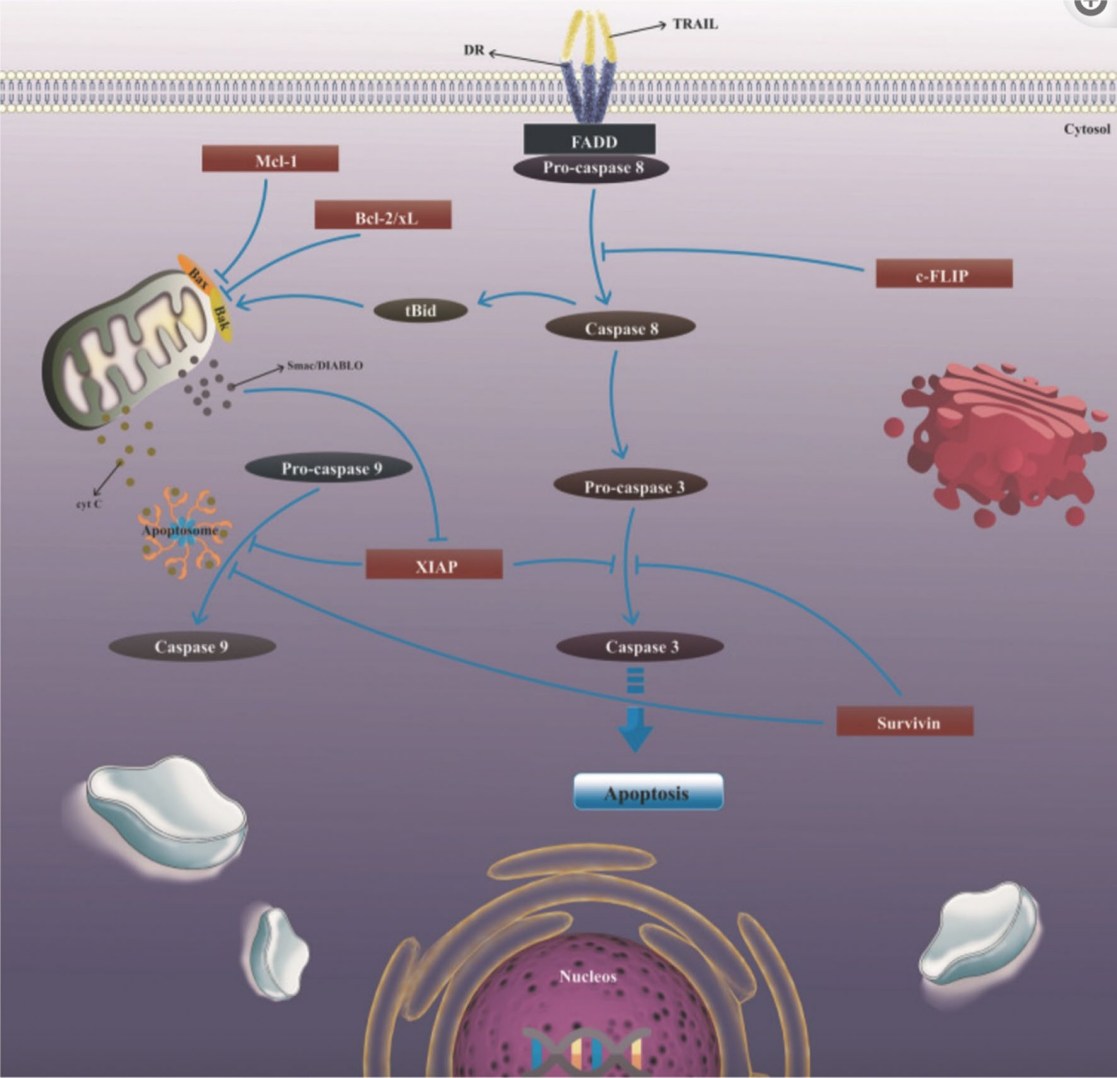
The mechanism of TRAIL-induced apoptosis in tumor cells. TRAIL (Tumor necrosis factor-related apoptosis-inducing ligand) induces apoptosis in tumor cells by binding to death receptors DR4 and DR5. This binding triggers the trimerization of the receptors and the subsequent translocation and activation of FADD (FAS-associating death domain-containing)

Another promising approach is the use of TRAIL (TNF-related apoptosis-inducing ligand) receptor agonists [99, 100] (Fig. 4). These agents activate the extrinsic apoptotic pathway by binding to death receptors on the cancer cell surface, leading to the formation of the death-inducing signaling complex (DISC) and caspase-8 activation. TRAIL receptor agonists have shown potential in inducing apoptosis selectively in cancer cells while sparing normal cells. Combining TRAIL agonists with chemotherapy, radiation, or immune checkpoint inhibitors has been explored to enhance their therapeutic efficacy and overcome resistance.

In addition to targeted therapies, immunotherapies have emerged as a potent strategy to modulate apoptosis in metastatic cancers. Immune checkpoint inhibitors, such as pembrolizumab (anti-PD-1) and nivolumab (anti-PD-1), have shown promise in reactivating immune responses against cancer cells [101, 102]. By blocking inhibitory signals that dampen T cell activity, these inhibitors can enhance the immune-mediated apoptosis of cancer cells. Combining immune checkpoint inhibitors with agents targeting apoptotic pathways, such as BH3 mimetics or TRAIL agonists, is an area of active research aiming to achieve synergistic effects and improve patient outcomes. Nanoparticle-based drug delivery systems offer another innovative approach to targeting apoptosis in bone metastatic lung cancer [103, 104]. These systems can enhance the delivery and bioavailability of apoptosis-inducing agents while minimizing systemic toxicity. Nanoparticles can be engineered to deliver a combination of therapeutic agents, providing a multifaceted attack on cancer cells by simultaneously targeting multiple pathways involved in apoptosis resistance.

### Experimental approaches and techniques in deciphering apoptotic signaling in bone metastatic lung cancer

The exploration of apoptotic signaling pathways in bone metastatic lung cancer heavily relies on in vitro models, serving as indispensable tools. Cultured cancer cells derived from metastatic lesions enable meticulous manipulation and observation of cellular responses under controlled conditions [105]. These models facilitate the identification of specific molecular alterations in apoptotic pathways, establishing a foundational understanding of the signaling events that drive apoptosis resistance. Utilization of diverse bone metastatic lung cancer cell lines, engineered to replicate genetic and phenotypic diversity observed in patients, provides valuable insights into apoptotic dynamics during various stages of metastasis.

In vivo models, employing animals such as mice, play a crucial role in translating in vitro findings into a physiologically relevant context. Orthotopic and metastatic models enable the replication of the intricate microenvironment of bone metastatic lung cancer, including interactions with the bone matrix, immune cells, and the vascular system. These models offer a holistic view of apoptotic signaling in a dynamic, living system, facilitating the assessment of therapeutic interventions and exploration of the microenvironment's impact on apoptosis modulation.

The study of apoptotic signaling in bone metastatic lung cancer has been revolutionized by advancements in molecular and imaging techniques. Molecular approaches, including polymerase chain reaction (PCR) and next-generation sequencing (NGS), enable the profiling of gene expression patterns associated with apoptotic pathways. Proteomic analyses, utilizing techniques like mass spectrometry, unveil post-translational modifications fine-tuning the apoptotic response. Advanced imaging technologies, such as fluorescence microscopy and positron emission tomography (PET), allow real-time visualization of apoptotic events within the complex microenvironment. This study utilized subtractive Cell-SELEX to identify a highly specific DNA aptamer, LP-16, which binds with high affinity to highly metastatic 143B cells but not to non-metastatic U-2 OS or normal cells, suggesting its potential as a molecular probe for diagnosing osteosarcoma metastasis [106]. Molecular imaging, in particular, provides a non-invasive means to monitor apoptotic changes longitudinally, offering valuable insights into the spatial and temporal dynamics of apoptosis in the metastatic context. The study investigated the efficacy of a PI3K inhibitor, buparlisib, in controlling bone metastasis of NSCLC using a xenograft model in mice. Results showed that buparlisib significantly inhibited tumor growth and osteoclast formation, as well as improved weight-bearing capacity, suggesting its potential as a therapeutic strategy to prevent skeletal damage associated with lung cancer bone metastasis [107].

Collectively, these experimental approaches and techniques provide a robust toolkit for researchers to comprehensively dissect apoptotic signaling in bone metastatic lung cancer [108]. The integration of findings from diverse methodologies allows for a nuanced understanding of the molecular intricacies governing apoptosis in the dynamic and challenging milieu of metastatic disease.

## Future and perspective

The treatment of bone metastatic lung cancer remains an exceptionally challenging and difficult endeavor due to several intrinsic and extrinsic factors associated with the disease. One of the primary difficulties lies in the heterogeneity of cancer cells within metastatic lesions. This heterogeneity not only pertains to genetic and epigenetic differences but also extends to variations in apoptotic signaling pathways, making it difficult to develop a one-size-fits-all treatment approach. The presence of subpopulations of cancer cells that exhibit resistance to apoptosis further complicates treatment efforts, as these cells can survive therapeutic interventions and lead to disease recurrence and progression. Another significant challenge is the unique and complex microenvironment of the bone. The bone microenvironment, which includes interactions with bone marrow stromal cells, osteoclasts, osteoblasts, and immune cells, provides a supportive niche for metastatic cancer cells. This environment is rich in growth factors, cytokines, and extracellular matrix components that not only facilitate cancer cell survival and proliferation but also contribute to therapeutic resistance by protecting cancer cells from apoptosis.

Furthermore, the dynamic nature of bone remodeling adds another layer of complexity. The continuous process of bone formation and resorption creates a fluctuating environment that can influence the behavior of metastatic cancer cells and their response to treatment. This aspect of bone biology requires therapeutic strategies that can adapt to or counteract these changes effectively. The blood–bone barrier also poses a significant obstacle to effective treatment. This barrier limits the delivery and penetration of therapeutic agents to metastatic sites within the bone, reducing the efficacy of systemic therapies. Additionally, the hypoxic conditions often found in the bone microenvironment can induce resistance to apoptosis and further complicate treatment efforts.

Given these challenges, future therapeutic strategies must adopt a multifaceted approach. Scientists should focus on developing targeted therapies that can address the specific molecular and cellular characteristics of bone metastatic lung cancer. This includes the continued development and refinement of BH3 mimetics, TRAIL receptor agonists, and immune checkpoint inhibitors. Combining these targeted therapies with conventional treatments such as chemotherapy and radiation, as well as novel approaches like nanoparticle-based drug delivery systems [109, 110], may enhance their efficacy and overcome resistance mechanisms. Moreover, personalized medicine approaches that utilize molecular profiling of individual tumors to guide treatment decisions are crucial. Understanding the unique genetic and epigenetic landscape of each patient's cancer can help tailor therapies that are more likely to be effective.

Another perspective that scientists need to embrace is the importance of targeting the tumor microenvironment. Therapies that disrupt the supportive interactions between cancer cells and the bone microenvironment, such as inhibitors of bone resorption or agents that modulate the immune microenvironment, hold promise in reducing metastatic growth and improving treatment outcomes. Lastly, leveraging emerging technologies such as CRISPR/Cas9 for gene editing and advanced imaging techniques for real-time monitoring of treatment responses can provide valuable insights and enhance therapeutic precision.

## Therapeutic implications and challenges

Current therapeutic strategies for bone metastatic lung cancer strategically exploit vulnerabilities within apoptotic pathways. The targeting of the Bcl-2 family, exemplified by small molecules like venetoclax, demonstrates promise by selectively inhibiting anti-apoptotic proteins, tipping the balance in favor of programmed cell death. Additionally, agents such as BH3 mimetics are designed to disrupt protein–protein interactions within the Bcl-2 family, sensitizing metastatic cells to apoptosis. In the extrinsic pathway, monoclonal antibodies targeting death receptors or ligands aim to amplify death signals, fostering apoptotic responses. Combining these strategies with conventional chemotherapeutics forms a multifaceted approach, synergistically enhancing the apoptotic susceptibility of metastatic cells.

Despite promising advances, the development of apoptosis-targeted therapies for bone metastatic lung cancer faces formidable challenges. For instance, while in vitro models provide valuable insights into apoptotic signaling pathways, they may oversimplify the complex tumor microenvironment and lack physiological relevance. Similarly, in vivo models, while more representative of the human condition, may have limitations in terms of species differences and the inability to fully recapitulate the human tumor microenvironment. Additionally, molecular and imaging techniques, while powerful tools, may suffer from inherent biases such as detection limits, signal amplification, and interpretation variability. Heterogeneity in apoptotic responses among metastatic cells poses a hurdle, as not all cells within a lesion may respond uniformly to therapy. Resistance mechanisms, including mutations or alternative survival pathways, can undermine the efficacy of apoptotic-targeted agents. The intricate crosstalk between apoptotic pathways and the tumor microenvironment further complicates therapeutic outcomes. Immunomodulatory effects induced by apoptotic-targeted therapies may either enhance or suppress the immune response, necessitating a delicate balance to optimize therapeutic benefits [111]. Additionally, systemic toxicities and off-target effects pose challenges, demanding precise delivery strategies to minimize adverse events.

The future of therapeutic interventions in bone metastatic lung cancer lies in innovative approaches transcending traditional paradigms. Personalized medicine, guided by molecular profiling of individual tumors, holds immense potential for tailoring apoptosis-targeted therapies. The synergy between apoptosis-targeted agents and immunotherapies emerges as a promising avenue, capitalizing on the immune system to eradicate apoptotic-resistant cells. Nanoparticle-based drug delivery systems offer a means to enhance drug bioavailability and reduce systemic toxicities, addressing challenges in treatment delivery [112, 113]. Furthermore, emerging technologies like CRISPR/Cas9 gene editing provide tools to manipulate apoptotic pathways directly within metastatic cells, overcoming intrinsic resistance mechanisms. The integration of apoptotic pathway modulators with existing treatment regimens represents a promising strategy for enhancing therapeutic outcomes in lung metastatic bone cancer. By targeting key regulators of apoptosis, such as the Bcl-2 family proteins and caspases, novel therapeutics can potentiate the apoptotic response in cancer cells, thereby inhibiting tumor growth and metastasis. For instance, small molecule inhibitors that selectively block anti-apoptotic proteins, such as Bcl-2 or Bcl-xL, have shown efficacy in preclinical models of cancer and are being evaluated in clinical trials for various malignancies [114, 115]. The study reveals that cancer cells resist apoptosis through BCL-2 family deregulation, but this resistance can be overcome by combining BTSA1.2 with Navitoclax, demonstrating synergistic efficacy in resistant cancers while sparing healthy tissues, offering a novel therapeutic strategy [114, 116]. Additionally, agents that activate pro-apoptotic pathways, such as BH3 mimetics or TRAIL receptor agonists, hold promise for inducing tumor cell death selectively. Moreover, combination therapies that target multiple nodes within the apoptotic pathway, or synergize with conventional treatments like chemotherapy or radiotherapy, offer the potential for enhanced efficacy and reduced drug resistance.

In conclusion, while current therapeutic strategies show promise in targeting apoptotic pathways in bone metastatic lung cancer, the field is not without its challenges. Overcoming limitations, understanding and circumventing resistance mechanisms, and embracing innovative trends will shape the next era of therapeutic interventions. As research progresses, the integration of these strategies into a comprehensive and personalized treatment approach holds the potential to redefine the therapeutic landscape for individuals grappling with the complexities of bone metastatic lung cancer.

## Discussion and conclusion

The investigation into apoptotic signaling pathways in bone metastatic lung cancer has unveiled a nuanced and dynamic terrain, providing crucial insights into the molecular intricacies dictating the destiny of metastatic cells. Dysregulation of apoptotic pathways, marked by perturbations in the Bcl-2 family, caspases, and regulatory proteins, emerges as a central player in apoptosis resistance during the metastatic cascade. The distinctive challenges posed by the tumor microenvironment, encompassing interactions with the extracellular matrix, immune cells, and angiogenic processes, further contribute to the intricacies of apoptotic dynamics in bone metastatic lung cancer. While current therapeutic strategies, notably those targeting the Bcl-2 family and employing apoptosis-inducing agents, exhibit promise, they confront hurdles such as the heterogeneity in apoptotic responses and the emergence of resistance. The trajectory of therapeutic interventions may pivot towards personalized medicine, synergistic amalgamations with immunotherapies, and inventive delivery systems, offering avenues to surmount extant challenges.

The imperative for ongoing research in comprehending apoptotic signaling pathways in bone metastatic lung cancer is accentuated by the unveiled intricacies and challenges. The heterogeneous nature of apoptotic responses among metastatic cells mandates a sophisticated understanding that transcends oversimplified generalizations. Specifically, addressing challenges in drug delivery, such as achieving sufficient concentrations at the tumor site while minimizing systemic toxicity, is crucial for clinical applicability. Moreover, considering patient-specific factors, such as genetic variations, tumor heterogeneity, and comorbidities, is essential for personalized treatment approaches. Additionally, discussing potential resistance mechanisms that may arise during therapy and strategies to overcome them would provide a more comprehensive understanding of the clinical landscape.

These include in vitro models to dissect molecular alterations, in vivo models to replicate physiological conditions, and molecular techniques like PCR and proteomics for comprehensive analysis. Future prospects entail refining personalized therapies, integrating immunotherapies, and advancing drug delivery systems. Molecular profiling, harnessed by advanced imaging technologies and emerging tools like CRISPR/Cas9, offers unprecedented opportunities to delve deeper into the complexities of apoptotic regulation, fostering a more comprehensive understanding of the disease. The amalgamation of multidisciplinary approaches, ranging from fundamental science to translational research, will further propel the field forward.

The revelations stemming from the scrutiny of apoptotic signaling in bone metastatic lung cancer bear profound implications for future clinical interventions. Personalized approaches, cognizant of the individual molecular profile of tumors, hold the potential for tailored and more efficacious therapies. The revelations stemming from the scrutiny of apoptotic signaling in bone metastatic lung cancer bear profound implications for future clinical interventions. Personalized approaches, cognizant of the individual molecular profile of tumors, hold the potential for tailored and more efficacious therapies. The synergistic interplay between apoptosis-targeted agents and immunotherapies presents an enticing prospect for augmenting treatment outcomes. Envisioning the clinical landscape, the evolution of targeted therapies, coupled with innovative delivery systems, holds promise for mitigating off-target effects and systemic toxicities. Moreover, the assimilation of emerging technologies, such as gene editing, introduces the prospect of directly modulating apoptotic pathways within metastatic cells.

In conclusion, the ongoing exploration of apoptotic signaling pathways not only enriches our comprehension of the molecular intricacies governing bone metastatic lung cancer but also propels us towards a future where personalized and precisely targeted interventions revolutionize clinical outcomes. The journey to unravel the complexities of apoptosis in metastatic disease is ongoing, and with each discovery, we approach transformative breakthroughs in the battle against this formidable adversary.

## Data Availability

No datasets were generated or analysed during the current study.
